# Effect of the Skill, Gender, and Kick Order on the Kinematic Characteristics of Underwater Undulatory Swimming in the Dorsal Position

**DOI:** 10.5114/jhk/168600

**Published:** 2023-10-11

**Authors:** Santiago Veiga, Xiao Qiu, Alfonso Trinidad, Burcu Ertas Dolek, Alfonso de la Rubia, Enrique Navarro

**Affiliations:** 1 Departamento de Deportes, Universidad Politécnica de Madrid, Madrid, Spain.; 2 Institute for Sports and Sport Science, University of Kassel, Kassel, Germany.; 3 Grupo de investigación AquaLab, Departamento de Educación y de Humanidades, Universidad Europea, Villaviciosa de Odón, Madrid, Spain.; 4 Sport Sciences Department, Ankara University, Ankara, Turkey.; 5 Departamento de Salud y Rendimiento Humano, Universidad Politécnica de Madrid, Madrid, Spain.

**Keywords:** performance, segmental kinematics, push start, backstroke

## Abstract

Backstroke swimmers display the greatest contribution of underwater kicking during the swimming race distances, but, surprisingly, there is little evidence of how kicking kinematics in the dorsal position should be performed. The aim of the present study was to examine the kinematic characteristics of competitive swimmers during underwater undulatory swimming in the dorsal position, with special attention to the swimmers’ gender, the level of skill, and kick order. Forty-one national-level swimmers (27 females and 14 males) were filmed from an underwater lateral view while performing a 25-m backstroke from a push start, and they were divided into fast and slow groups according to their kicking velocity. Direct linear algorithms were employed to reconstruct the two-dimensional kinematic characteristics of the first and final kicks of the underwater section. There were no differences between males and females in kicking performance when data were normalised to the swimmers’ height. However, swimmers in the fast-kicking group were distinguished by a greater kicking frequency (η^2^: 0.15) and specific segmental kinematics related to a lower knee range of motion. Swimmers decreased kicking velocity (η^2^: 0.47) in addition to the kicking frequency (η^2^: 0.31) and length (η^2^: 0.16), but increased the kicking amplitude (η^2^: 0.11) between the first and the final kicks. Changes in kicking segmental kinematics were more related to modification in body orientation during the underwater trajectory than to the kicking motion itself. These results provide the first solid evidence of how swimmers should kick for better performance in dorsal underwater swimming.

## Introduction

The underwater undulatory swimming (UUS) technique is the fastest means of human locomotion in the water (Hochstein et al., 2012). This represents a key aspect in competitive swimming, where athletes try to extend the underwater swimming sections of the race in order to achieve a net gain of overall velocity in comparison to the swimming at the water surface. The only constraints for that purpose are the World Aquatics regulations that limit the maximum distance swimmers can travel before breaking the water surface to 15 m from the starting and/or the turning wall.

Previous evidence on international level competitions has revealed how small changes in underwater distances travelled by swimmers (and their average velocities) can have a meaningful impact on the overall swimming race performance (Veiga et al., 2016). Moreover, international level swimmers have shown a greater ability to maintain underwater performance over successive laps compared to national level swimmers who decrease their underwater distances or velocities at the end of races (Veiga and Roig, 2016). Considering the decrease in forward velocity occurring from the instant the swimmers leave the starting and/or the turning wall (Gonjo and Olstad, 2020), competitors must use individual strategies in their kicking kinematics for the duration of their underwater sections in relation to their surface swimming velocity (Veiga and Roig, 2017) and their apnoea-related fatigue state (Veiga et al., 2022b).

In order to maximise forward velocity during underwater race sections, a recent systematic review concluded that faster swimmers in UUS obtained a greater kicking frequency than slower swimmers, but a smaller kicking amplitude (Veiga et al., 2022a). This was coupled with differences observed in the body undulation motion, where faster swimmers displayed a more symmetrical movement of the joint along the body axis (Hochstein and Blickhan, 2014), a lower amplitude in the movement of the arm-hand region, and a greater peak of angular velocity on the chest, lower trunk, thigh, and shank segments (Tanaka et al., 2022) during kicking. All of these differences resulted in a faster vertical velocity of the toes during both the upkick and the downkick for faster swimmers in UUS (Higgs et al., 2017).

Male swimmers have been generally reported as better kickers than their female counterparts, as they present faster velocities (Wądrzyk et al., 2019) and longer underwater distances when in competition (Veiga et al., 2014). However, gender differences in UUS performance could be related to the anthropometric differences between males and females. Indeed, for surface swimming (both upper and lower limbs), the greater height, arm span, and hand size usually presented by males have been positively correlated with swimming velocity, specifically with longer stroke lengths (Seifert et al., 2010). Conversely, no general differences in the stroke frequency have been typically observed when comparing the same relative swimming intensities for both genders (Pelayo et al., 1996).

All the reported evidence on the kicking motion of competitive swimmers has been based on observations during the ventral kicking technique. There are few studies that examine the kinematic determinants of UUS in the dorsal position, despite backstroke being the stroke by which competitive swimmers travel the longest distance underwater (Morais et al., 2019). Only data presented in conference communications have revealed no meaningful differences in kicking variables or kicking performance between UUS performed in the ventral or the dorsal position (Alves et al., 2006; Arellano et al., 1999;Demirkan et al., 2023). Therefore, the aim of the present study was to examine the kinematic characteristics of competitive swimmers when performing UUS in the dorsal position, with special attention to changes along the underwater sections and to the swimmers’ gender and the level of skill. It was hypothesised that faster swimmers in dorsal UUS would present greater kicking frequencies at the end of underwater sections coupled with differences in segmental kinematics related to the body undulation. Also, gender differences in dorsal UUS would be dependent on the anthropometrics.

## Methods

Forty-one national-level swimmers, 27 females (age: 16.4 ± 1.42 years; body height: 1.76 ± 0.06 m; body mass: 61.33 ± 7.35 kg) and 14 males (age: 15.4 ± 1.02 years old; body height: 1.61 ± 0.04 m; body mass: 51.13 ± 6.85 kg), participated in the present study. All had a competitive experience of at least four years and accredited personal best times that allowed them to participate in national championships. According to the standardisation of swimming performances (Ruiz-Navarro et al., 2021), participants belonged to level two and level three ties. Written consent was obtained from all participants and their tutors before the commencement of the experiment, and the Universidad Politécnica de Madrid Research Ethics Committee approved the experimental procedures (Reference: 2020–080 on 19 November 2020). The study was performed according to the Declaration of Helsinki.

Two sequential video cameras (JVC GY-DV500E) filmed the trials from a lateral-underwater view at 50 Hz. Cameras were stationary, with their optical axis perpendicular to the swimming direction and located at 3 and 8 m, respectively, from the starting wall. The distance from the cameras to the centre of the swimmers’ lane was approximately 8 m. Before the swimming trials, a rectangular calibration frame (4 m length x 2 m width) containing eight control points was located in the centre of the swimmers’ lane and was filmed for calibration purposes. Before the recordings, black markers of 25 mm diameter and consisting of adhesive tape were located in specific body landmarks of the right side of the swimmers’ body (styloid process of the ulna [wrist], greater tubercle of the humerus [shoulder], great trochanter [hip], lateral epicondyle of the femur [knee], lateral malleolus [ankle], and the epiphysis of the fifth metatarsal [toe]). This was done to assist in the further analysis of underwater footage. After a standardised warm-up in water of approximately 1000 m and in an indoor 50-m swimming pool with water at 27°C, swimmers performed one backstroke repetition of 25 m at maximal velocity from a push start. This means that swimmers began with an in-water starting position, where both feet were situated approximately 1 m below the water surface and with swimmers facing the starting wall (Trinidad et al., 2022). No specific constraints were given to swimmers in relation to the underwater or surface swimming distribution on the 25-m effort apart from World Aquaticsregulations (maximum 15 m of underwater swimming from the starting wall).

An experienced observer manually digitised the swimming trial footage using Kinovea 0.9.5 software (Joan Charmant& Contrib., kinovea.org). In line with the study aims, the first and final complete undulations during the underwater kicking segment of each 25-m swimming trial were identified. For each swimmer, the wrist, shoulder, hip, knee, ankle, and toe joint centres were marked at the upkick and downkick positions. The upkick position was defined as the highest point, whereas the downkick was the lowest point of the swimmer’s toe during body undulations. Two-dimensional direct linear transformation algorithms (Abdel-Aziz and Karara, 2015) were used to convert the screen coordinates in real-space coordinates. Reconstruction error was computed using eight control points in the calibration frame not used for calibration purposes, as explained in detail by Veiga et al. (2023). In addition, the intra-observer reliability was tested by repeatedly digitizing the same swimming trial in twenty non-consecutive times with a coefficient of variation lower than 1.5%.

**Figure 1:**
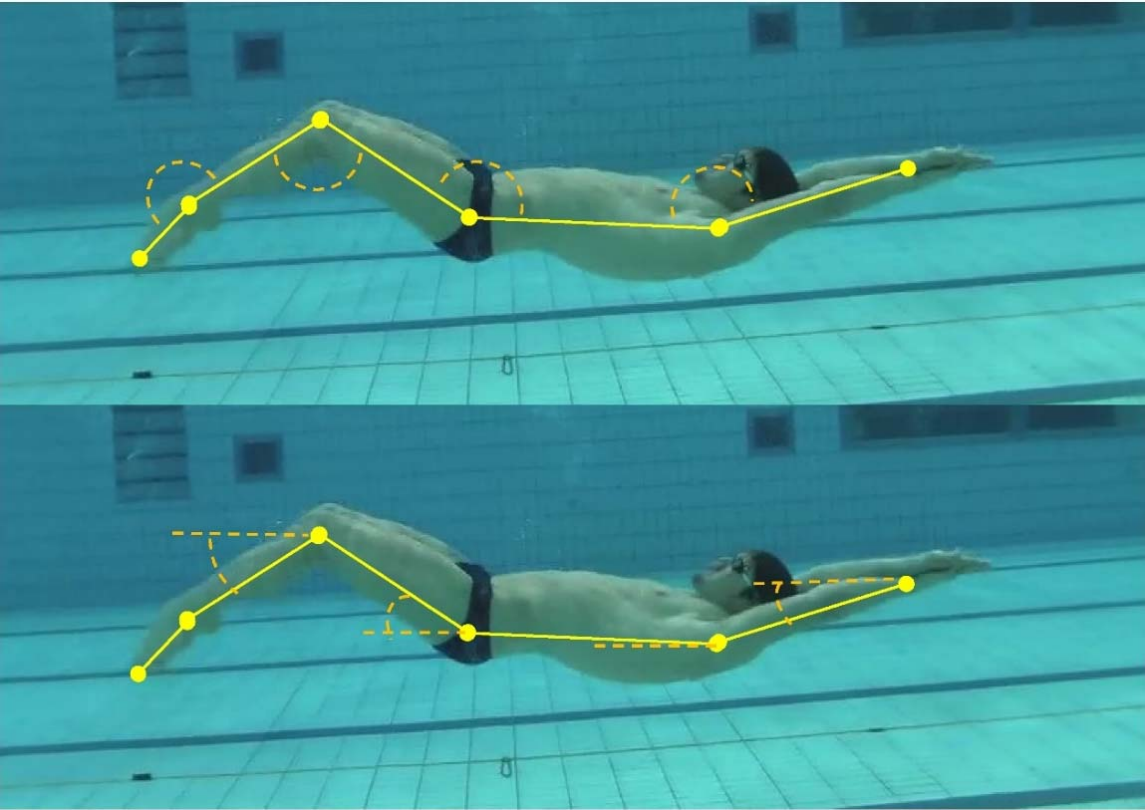
Atypical down-kick position for UUS in the dorsal position for competitive swimmers with schematic joint angles (above) and segment inclinations (below) in yellow colour.

The horizontal displacement travelled by the swimmers’ hip during complete underwater kicking (from a downkick to the following downkick position) was computed as kicking length in metres. On the other hand, the vertical displacement of the swimmers’ toe from the upkick to the downkick position was measured as kicking amplitude (m). The inverse of the time in seconds employed to perform the complete underwater kick was expressed as a kicking frequency (Hz). Finally, UUS performance was measured as kicking velocity (m/s) resulting by multiplying kicking length and the kicking frequency. All these kinematic variables were normalised to the swimmers’ height. The segmental kinematics of UUS were also computed by calculating i) the shoulder, hip, knee, and ankle angles (in degrees); ii) the upper limbs, trunk, thigh, shank, and feet segment inclinations in relation to the horizontal angle; and iii) the relative positions of the joints to the hip centre (Ikeda et al., 2021), according to Figure 1.

The descriptive results of all variables are expressed as mean values ± SD. Repeated measures MANOVA (multivariate analysis of variance) was used to compare the biomechanical differences between the first and final dorsal kicks according to gender (male vs. female) and skill (fast vs. slow group). The skill levels were distinguished based on swimmers’ average kicking velocity regardless of gender: the faster 50% average kicking velocity of swimmers was assigned to the fast group, and the rest of the swimmers were then assigned to the slow group. Effect sizes (partial eta square, *η*^2^) were calculated to determine the meaningfulness of differences categorised as small (0.01), medium (0.06), or large (0.14) (Knudson, 2009). All statistical procedures were conducted using IBM SPSS Statistics 25.0 (IBM Corp, Armonk, NY, USA). Statistical significance was set at *p*< 0.05.

## Results

### Kicking Variables

The kicking velocity of competitive swimmers in the dorsal position decreased by 13.6% between the first and the final kick of the underwater section (Table 1). Changes in velocity were accompanied by a decrease in the kicking frequency (8.1%) and, in a lesser degree, kicking length (6.1%). On the other hand, the kicking amplitude increased by 20.0% in the final kick. These modifications occurred regardless of the swimmers’ gender (kick × gender, *p*> 0.05) or level of skill (kick × skill, *p*> 0.05). Male swimmers presented greater absolute kicking velocities and kicking frequencies than their female counterparts, although no differences were observed between genders (*p* > 0.05) when values were normalised to the swimmers’ height (Table 1). In relation to the skill level of swimmers, faster athletes (1.66 ± 0.16 vs. 1.44 ± 0.11 m/s of slow group; F_1_ = 34.18, *p* = 0.001, *η*^2^ = 0.48) presented a greater kicking frequency (2.30 ± 0.31 vs. 2.11 ± 0.23 Hz; F_1_ = 6.48, *p* = 0.02, *η*^2^ = 0.15), whereas no differences in kicking length (F_1_ = 2.40, *p* = 0.13, *η*^2^ = 0.06) or the kicking amplitude (F_1_ = 0.12, *p* = 0.73, *η*^2^ = 0.01) were observed. These skill effects on the kicking frequency were also present when values were normalised to the swimmers’ height.

**Table 1:** Kicking variables of national level swimmers during dorsal UUS.

	First kick	Last kick	Kick effect (η^2^)		Gender effect (η^2^)	
			Raw data	Normalized	Raw data	Normalized
Kicking velocity (m/s)						
Males	1.78 ± 0.20	1.59 ± 0.19	0.47*	0.30*	0.37*	0.00
Females	1.57 ± 0.21	1.32 ± 0.13				
Kicking frequency (Hz)						
Males	2.45 ± 0.25	2.22 ± 0.24	0.31*	0.48*	0.16*	0.02
Females	2.16 ± 0.31	2.00 ± 0.23				
Kicking length (m)						
Males	0.73 ± 0.06	0.72 ± 0.07	0.16*	0.16*	0.01	0.07
Females	0.73 ± 0.09	0.66 ± 0.09				
Kicking amplitude (m)						
Males	0.36 ± 0.08	0.43 ± 0.16	0.11*	0.10*	0.01	0.01
Females	0.36 ± 0.09	0.40 ± 0.17				

* *p*< 0.05

### Segmental Kinematics

**Figure 2:**
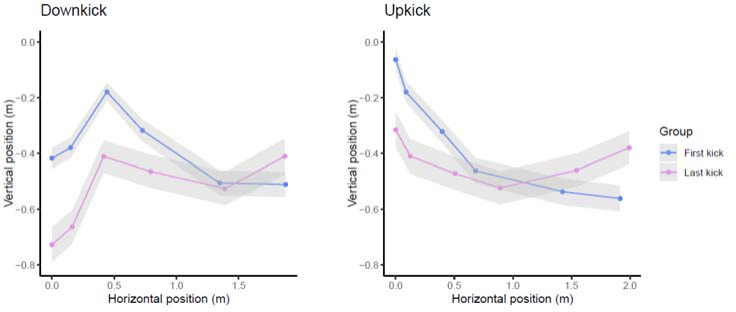
Schematic representation of the position of selected joint centres by competitive swimmers during UUS in the dorsal position.

Changes in the kicking variables were accompanied by changes in the segmental body position from the first to the final kick (Table 2). As observed in Figure 2, the position of joint centres in relation to the hip position revealed large changes during the underwater section, highlighting a greater maximal body sagittal amplitude during the first kick, especially in the upkick position (0.53 ± 0.11 m vs. 0.37 ± 0.09 m in the downkick). During kicking motion (from the downkick to the upkick), the ankle and shoulder joint centres elevated, whereas the knee and hip joint centres descended.

**Table 2:** Segmental kinematics in meters (vertical positions of joint centres relative to the hip) of national level swimmers during UUS in the dorsal position.

First kick	Last kick		Kick effect (η^2^)
−0.06 ± 0.07	–0.20 ± 12		0.43*
0.28 ± 0.08	0.11 ± 0.10		0.64*
0.14 ± 0.05	0.05 ± 0.07		0.44*
0.14 ± 0.04	0.05 ± 0.06		0.63*
–0.19 ± 0.07	–0.06 ± 0.10		0.44*
–0.07 ± 0.07	0.06 ± 0.06		0.62*
–0.19 ± 0.10	0.05 ± 0.12		0.66*
–0.10 ± 0.11	0.14 ± 0.06		0.74*
	
0.36 ± 0.09	0.39 ± 0.09		0.02
0.52 ± 0.11	0.25 ± 0.07		0.80*

* *p*< 0.05

**Figure 3:**
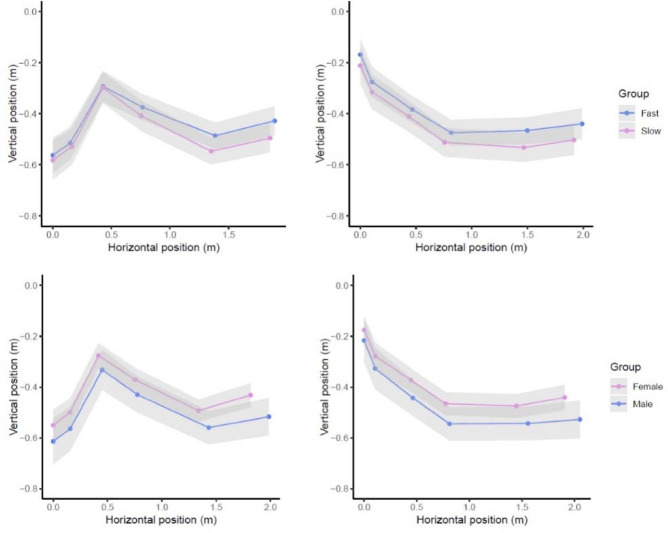
Schematic representation of the body joint centres position in the first and the last kick of UUS in the dorsal position by competitive swimmers.

No differences were observed for any of the segmental variables between male and female swimmers, although some effects were observed according to the swimmers’ level of skill. Faster swimmers presented a lower position of the knee relative to the hip in the downkick (F_1_ = 5.30, *p* = 0.03, *η*^2^ = 0.13) and a higher position of the wrist relative to the hip in both the downkick (F_1_ = 5.32, *p* = 0.03, *η*^2^ = 0.13) and upkick (F_1_ = 4.58, *p* = 0.04, *η*^2^ = 0.11) positions. In addition, the vertical displacement of the knee joint centre during kicking was lower for the fast group of swimmers (F_1_ = 5.74, *p* = 0.02, *η*^2^ = 0.13). The average positions of the joint body centres by gender and the skill level can be found in Figure 3.

### Angular Kinematics

The inclinations of the body segments also showed large changes between the first and the final kick of dorsal UUS (Table 3), with no statistical differences between genders (*p*> 0.05). However, the faster swimmer group in the present study displayed lower thigh inclinations to the horizontal angle at the downkick (F_1_ = 6.96, *p*< 0.001, *η*^2^ = 0.16). For the joint angles, no differences were observed between the first and the final kick of UUS except a greater ankle angle at the downkick of the first kick and greater hip angles in the upkick of the final kick (Table 3). Male and female swimmers did not present different joint angles during the downkick and upkick positions, in the same line as the fast and slow groups. However, faster swimmers displayed a greater knee angle at the downkick (F_1_ = 4.25, *p*< 0.05, *η*^2^ = 0.11) and a lower knee range of motion (ROM) during kicking (F_1_ = 4.10, *p*< 0.05, *η*^2^ = 0.10).

**Table 3:** Angular kinematics of national level swimmers during UUS in the dorsal position.

	First kick	Last kick	Kick effect (η^2^)
Leg (°)			
	35 ± 9	44 ± 21	0.15*
	−25 ± 8	–9 ± 12	0.54*
Thigh (°)			
	–25 ± 12	–8 ± 10	0.46*
	–26 ± 9	–8 ± 9	0.67*
Trunk (°)			
	–17 ± 8	–7 ± 9	0.41*
	–6 ± 6	5 ± 5	0.60*
Upper limbs (°)			
	0 ± 6	13 ± 6	0.52
	–3 ± 7	9 ± 9	0.35*
Ankle (°)			
	159 ± 12	151 ± 15	0.13*
	151 ± 11	151 ± 11	0.00
Knee (°)			
	118 ± 12	121 ± 10	0.03
	175 ± 5	173 ± 8	0.02
Hip (°)			
	166 ± 11	171 ± 8	0.07
	159 ± 12	166 ± 10	0.12*
Shoulder (°)			
	163 ± 9	159 ± 10	0.10*
	173 ± 4	171 ± 8	0.03

* *p*< 0.05

## Discussion

Despite the increasing interest of the swimming community in UUS techniques, there is no clear evidence on the kinematic characteristics of competitive swimmers during undulatory kicking in the dorsal position. In the present study, changes in the dorsal kicking motion were observed from the beginning to the end of underwater sections, and faster swimmers were characterised by a greater kicking frequency coupled with specific segmental differences in the upkick and downkick positions. In addition, surprisingly, no gender differences were detected in the kicking velocity and kinematic variables when data were normalised to the swimmers’ height.

### Kinematic Characteristics of Dorsal UUS

Kicking velocities in the dorsal position were in line with values from international-level swimmers (Arellano et al., 1999), but lower than those previously reported for national-level swimmers in ventral kicking, especially in the first kick after the push start (Veiga et al., 2023). The reason may be that swimmers aimed at a deeper trajectory after the wall push-off in order to avoid their feet breaking the water surface in the upkick position. As a consequence, they adopted more vertical body positioning on the first kick with maximum inclinations of the leg, thigh, and trunk segments and the maximum vertical amplitude of the body position at that point (Figure 2). Kicking frequencies (all groups above two kicks per second) were greater than typically reported in the ventral position (Veiga et al., 2023). This was coupled with shorter kicking amplitudes and kicking length values for dorsal kicking, unlike previous evidence over 15 m (Arellano et al., 1999) or 25 m (Alves et al., 2006), where no meaningful kinematic differences between dorsal and ventral undulatory kicking were detected. Indeed, the theoretical drag levels of the dorsal versus the ventral body position should not justify large differences in kicking performance between the two body positions (Marinho et al., 2011).

During the kicking motion, swimmers in the present research increased the vertical displacement of joint centres from a caudal to a distal direction (shoulder to ankle), at a similar magnitude as previously reported (Gavilán et al., 2006). The hip angle decreased and the shoulder angle increased from the downkick to the upkick position, with a similar angular displacement of body segments as presented in ventral kicking (minimum oscillation of the thigh and the trunk, and maximum oscillation of the leg segment) (Veiga et al., 2023). However, dorsal kicking also revealed some specific features in relation to ventral kicking. At the downkick, swimmers in the dorsal position displayed greater shoulder flexion and lower knee flexion than previously reported for national- and international-level swimmers in ventral kicking (Arellano et al., 2003; Veiga et al., 2023).

This resulted in greater shoulder ROM while kicking, but lower ROM of the knee joint compared to what was reported for ventral kicking, which would suggest greater upper-body oscillation of dorsal versus ventral kicking, in line with Arellano et al. (2003).

### Gender and Skill Effect on UUS Kinematic Characteristics

One of the main observations of the present research was that faster swimmers performing UUS in the dorsal position presented a greater kicking frequency than slower swimmers, with no differences in the kicking amplitude or kicking length. This seems to be a key finding considering the large sample of the present study and the lack of agreement in previous studies. For example, our results are in line with those from Arellano et al. (2003) who found greater kicking frequencies, but a similar kicking amplitude and kicking length between international- and national-level swimmers in ventral kicking. On the other hand, our data disagree with kicking length differences observed between a fast and a slow group of regional level swimmers (Tanaka et al., 2022). Interestingly, the existing evidence of the role of skill in swimming kinematics has traditionally conferred stroking length as the discriminant variable between faster and slower swimmers, regardless of the stroke (Barbosa et al., 2010). However, the fact that UUS during swimming starts occurs during short periods at maximum intensity could highlight the role of kicking frequency.

Faster swimmers also presented specific differences in the segmental kinematics that seemed to be linked to better body undulation movement. In the downkick, their knee angle was greater, their knee position relative to the hip was lower, and their thigh inclination in relation to the horizontal angle was lower. This was in line with previous evidence recommending lower angles of the attack and lower ROM for the thigh segment (Houel et al., 2013; Ikeda et al., 2021), and also a decrease in the maximum knee flexion (Arellano et al., 2003) during UUS. In addition, the hip position relative to the wrist was also lower in both the downkick and the upkick for the fast group. All these aspects could be explained by greater lower trunk mobility and ROM typically observed in faster swimmers (Ikeda et al., 2021; Tanaka et al., 2022) that could assist in a more symmetrical body undulation (Hochstein and Blickhan, 2014).

Another key finding of the present research was the lack of gender differences when kicking data were normalised to the swimmers’ height. Male swimmers did not present faster kicking velocities or kicking frequencies than females, in line with the lack of differences in the segmental kinematics. Previous studies (Wądrzyk et al., 2019) reported greater kicking length and amplitudes for male compared to female competitive swimmers in ventral UUS, but similar values of kicking frequency. In the same line, general differences in swimming velocity (both upper and lower limbs) between gender were mainly explained by differences in distance per stroke, which were attributed to the greater anthropometrics (height, segment length, etc.) (Pelayo et al., 1996) and strength levels of male swimmers (Seifert et al., 2010). However, results of the present study outlined that the gap in kicking performance was explained by differences in body size (males: 1.76 ± 0.06 m vs. females: 1.61 ± 0.04 m) rather than other factors. A similar kicking motion (Figure 3) and expected similar propelling efficiency (Zamparo, 2006) of male and female swimmers seem to explain the lack of further differences.

### Changes in Dorsal UUS Characteristics from the First to the Final Kick

The kinematic characteristics of dorsal UUS presented clear modifications from the beginning to the end of the underwater sections. Kicking velocity in the final kick underwater was approximately 14% slower than the first kick, and there was a concurrent decrease in the kicking frequency and length (Table 1). The magnitude of changes from the first to the final kick was similar to that reported in the underwater section of ventral kicking (Veiga et al., 2023), but no differences in the kinematic mechanisms were detected between genders. Previous studies hypothesised on the role of the strength level of males and females to explain differences in kicking frequency (Arellano et al., 2003). However, the present results confirm the importance of anthropometrics in UUS kinematic differences rather than swimmers’ gender, as no differences in the kicking evolution were detected when variables of males and females were normalized to body height. Interestingly, kicking amplitude values increased in the final kick underwater and showed an opposite tendency to kicking variables. In relation to the skill of swimmers, the evolution of kicking variables did not display differences between the faster and slower groups, and faster swimmers presented greater kicking velocities in both the first and final kicks. Previous studies revealed a better ability of faster swimmers to maintain velocities, both underwater (Veiga and Roig, 2016) and on the surface (Hellard et al., 2008), between successive swimming laps. However, this was probably related to better coping of faster swimmers with fatigue (Figueiredo et al., 2011). In the present study, where the duration of the underwater start section averaged around 4 s from the wall push-off, changes from the first to the final kick were probably related to the hydrodynamic drag rather than the accumulation of fatigue.

Overall, changes in kicking variables at the end of underwater sections seemed to be more related to the body orientation than the kicking movement itself. Swimmers presented a greater hip angle at the upkick position of the final kick (Table 3), but no further differences in the joints’ angular kinematics were detected. Large differences observed in the relative position of joint centres to the swimmers’ hip from the first to the final kick (Table 2) confirmed changes in the body orientation of a greater magnitude than observed in ventral kicking (Veiga et al, 2023). For example, the ankle, knee, shoulder, and wrist joints increased or decreased their relative positions to the hip by more than 10 cm at the downkick from the first to the final kick (Table 2). These data resulted in a maximal vertical amplitude of body joint positions during the first dorsal kick, and suggested different underwater trajectories of ventral versus dorsal kicking not previously reported.

## Limitations

The present study has some limitations that should be acknowledged to better interpret the results. During the underwater sections, only two kicking cycles (first and the last kick) were selected for kinematic analysis at two discrete positions (downkick and upkick) of the entire kicking cycle duration. In addition, the mechanic model of the swimmers’ body did not account for the upper and lower trunk segments that could provide further information about the body undulation movements. Future studies examining the kinematic evolution of UUS variables in the dorsal position through the entire underwater trajectories could help understand how to minimize the decrease in kicking velocity at the end of underwatersections.

## Conclusions

The present results provide the first solid evidence on how swimmers should kick for better performance in dorsal underwater swimming. Kicking frequency (not kicking length) was the distinguishing variable between the swimmers’ level of skill when performing UUS in the dorsal position from a push start. In addition, faster swimmers presented specific segmental differences related to a lower knee ROM when kicking. Male and female swimmers displayed similar levels of kicking performance when variables were normalised to the swimmers’ height. These similar performances were outlined by similar segmental kinematics at both the first and final kicks of the underwater sections. Kicking kinematics in the dorsal position showed specific characteristics versus ventral kicking, as changes in the body alignment seemed to be greater with a higher vertical amplitude of body joints (especially in the first kick) and greater upper-body oscillation during kicking.
